# PLETHORAs shape Arabidopsis phyllotaxis through modulation of patterning robustness and accelerated inflorescence development

**DOI:** 10.1111/nph.70620

**Published:** 2025-10-11

**Authors:** Merijn Kerstens, Freek van der Klugt, Hugo Hofhuis, Ben Scheres, Viola Willemsen

**Affiliations:** ^1^ Cluster of Plant Developmental Biology, Cell and Developmental Biology Wageningen University & Research Droevendaalsesteeg 1 6708 PB Wageningen the Netherlands

**Keywords:** *Arabidopsis thaliana*, chirality, DAP‐seq, inflorescence meristem, phyllotaxis, PLETHORA, RNA‐seq, torsion

## Abstract

- Phyllotaxis is the arrangement of lateral organs on a stem axis, which in Arabidopsis follows a spiral pattern. We previously described that loss of three PLETHORA (PLT) transcription factors shifts the phyllotactic spiral to novel metastable patterns, but the mechanism behind these shifts remained unclear.
- In this study, we aimed to fill this knowledge gap by performing a detailed analysis of phyllotaxis in *plt* rosettes, inflorescences, and meristems. We supplement our quantitative measurements with transcriptomic profiling of *plt3 plt7* meristems, genome‐wide *in vitro* binding assays, and an EMS enhancer screen in *plt3 plt5 plt7*.
- Contrary to earlier beliefs, primordium positioning at the inflorescence meristem is only subtly noisier in *plt3 plt5 plt7*, which we attribute to loss of PLT‐controlled regulation of auxin and cytokinin networks. However, the meristematic patterning defects alone cannot explain the large deviations from the spiral pattern in mature mutant inflorescences. Instead, accelerated inflorescence development of *plt3 plt5 plt7* generates larger patterning deviations through the joint action of meristem chirality and stem torsion. We demonstrate the importance of the relationship between chirality, torsion, and internode length in tissue‐twisting mutants.
- We conclude that shoot meristematic PLTs provide robustness to primordium patterning and phyllotaxis through the integration of developmental processes during bolting.

## Introduction

The nonrandom position of lateral organs on a stem(‐like) axis is called phyllotaxis. In the great majority of plants including Arabidopsis (*Arabidopsis thaliana* (L.) Heynh.), leaves and/or flowers are positioned in a spiralling fashion with the divergence angle between two successive organs approaching *c*. 137.5°, the ‘golden angle’ (Kuhlemeier, 2007). From a vertical perspective, lateral organs are separated from each other through the growth of internodes, which together with circumferential patterning shapes aboveground plant architecture across the plant kingdom.

Lateral organ primordia are initiated in the shoot apical meristem (SAM) at the top of the plant. The SAM is a highly organised structure consisting of a stem cell niche in the central zone (CZ), around which leaves and/or flowers arise sequentially in the peripheral zone (PZ) surrounding the CZ. The stem cell pool is maintained through a regulatory feedback loop between CZ‐localised CLAVATA3 (CLV3) peptide and stem cell‐promoting factor WUSCHEL (WUS), which is expressed underneath the CZ in the organising centre (OC) (Brand *et al*., 2000; Schoof *et al*., 2000). In Arabidopsis, lateral organ initiation occurs distinctly in the vegetative (rosette) and generative (inflorescence) phases. In the rosette, the cotyledons and first two true leaves arise simultaneously at a *c*. 180° angle, after which subsequent leaves gradually convergence on the golden angle through damped oscillations (Mündermann *et al*., 2005; Smith *et al*., 2006). During the vegetative phase, internodes are short due to the mitotic inactivity of the so‐called rib zone below the OC, giving rise to a typical rosette habit. After floral transition, in which the SAM transforms into the inflorescence meristem (IM), cells in the rib zone are activated, leading to the formation of internodes and an inflorescence (Jacqmard *et al*., 2003). The spiral pattern of the rosette leaves is thus propagated to flower primordia, and thereafter flowers on the bolt.

It is firmly established that auxin signalling and transport through the polar efflux carrier PIN‐FORMED1 (PIN1) is the pivotal process underlying pattern formation at the shoot apex. Inhibiting PIN1‐mediated efflux with *N‐*1‐naphthylphthalamic acid (NPA) or through mutagenic approaches causes perturbations in SAM leaf patterning and IMs devoid of flower primordia (Okada *et al*., 1991; Reinhardt *et al*., 2000; Guenot *et al*., 2012). Nevertheless, the exact roles of levels, fluxes, and temporal integration remain disputed. In the classical model (Reinhardt *et al*., 2003; Heisler *et al*., 2005; de Reuille *et al*., 2006; Jönsson *et al*., 2006; Smith *et al*., 2006), feedback mechanisms would cause repolarisation of PIN carriers towards a local maximum, thereby accumulating more auxin and depleting the surrounding area. The depletion would restrict the formation of successive maxima to specific positions at the SAM and IM, generating the phyllotactic spiral. More recently, it was found that auxin maxima travel faster from the CZ than cells divide radially, and that PIN polarity reversal only occurs from the seventh flower primordium (P_7_) onwards (Galvan‐Ampudia *et al*., 2020), which is incompatible with the former model (Reinhardt *et al*., 2003; Heisler *et al*., 2005; de Reuille *et al*., 2006; Jönsson *et al*., 2006; Smith *et al*., 2006). An additional layer of overlooked complexity in both models is that cytokinin inhibitory fields also contribute to phyllotactic regularity (Besnard *et al*., 2014).

The initial position of organ primordia at the SAM/IM can be adjusted post‐initiation through twisting of the stem. It was shown that mutations in *CELLULOSE SYNTHASE INTERACTING 1* (*CSI1*) and *SPIRAL2* (*SPR2/TORTIFOLIA1*) cause oblique cortical microtubule arrays, leading to consistent counterclockwise torsion to resolve local mechanical conflict (Landrein *et al*., 2013; Verger *et al*., 2019). Torsion could then displace *c*. 137.5° divergence angles to smaller or larger values, depending on whether an IM produced primordia in a clockwise or counterclockwise fashion, respectively (Landrein *et al*., 2013). This shows that internodes do not only affect vertical patterning, but circumferential patterning as well.

We previously showed that the three PLETHORA (PLT) transcription factors expressed in the SAM and IM influence phyllotaxis (Prasad *et al*., 2011; Pinon *et al*., 2013). In the *plt3 plt5 plt7* triple mutant, the spiral arrangement of flowers on the inflorescence stem shifted noticeably to alternate decussate (90°–90°) or distichous patterns (180°–180°), although not consistently between experiments. Moreover, the transition of rosette leaves from a decussate to spiral arrangement was delayed to leaves 3 and 4 (Prasad *et al*., 2011). PLT activity in the CZ was required to rescue the triple mutant phenotype, which exhibited reduced auxin signalling due to compromised auxin biosynthesis (Pinon *et al*., 2013). However, it remained unclear why divergence angles switched to metastable 90° or 180° arrangements and whether auxin biosynthesis was the central process regulated by PLTs.

In this study, we used detailed phenotyping methods to reassess how PLTs regulate phyllotaxis. We discovered that inflorescence phyllotaxis in the *plt3 plt5 plt7* mutant does not adopt novel metastable patterns, but rather decreases the regularity of the spiral configuration. Through RNA‐ (RNA‐seq) and DAP‐sequencing (DAP‐seq), we identified that PLTs directly regulate known primordium positioning genes involved in auxin and cytokinin signalling, and we describe a newly identified weak *pin1* allele that requires the loss of PLTs to display an organ initiation phenotype. However, closer inspection revealed that primordium positioning defects *in plt3 plt5 plt7* and IMs could not explain the divergence angles on the inflorescence. Instead, the *plt* triple mutant bolts earlier and grows faster, resulting in longer internodes which, together with inherent counterclockwise stem torsion, displaces divergence angles from their initial spiral arrangement in a chirality‐dependent fashion. We further confirmed the impact of stem torsion on phyllotaxis by analysis of twisting mutants. We thus redefine the role of PLTs in phyllotaxis as providing patterning robustness to the SAM/IM.

## Materials and Methods

### Plant materials and growth

*plt3 plt7*, *plt3 plt5 plt7*, *plt3 plt4 plt5 plt7*, and *plt3 plt5 plt7‐t* have been described previously (Prasad *et al*., 2011; Kerstens *et al*., 2021). *spr2‐2* (CS6549), *tor2* ^*T56I*^ (CS68879), and *tor2* ^*S178Δ*^ (CS68881) were provided by the Arabidopsis Biological Resource Center. Seeds were germinated on a ½‐strength Murashige & Skoog (½MS) medium (1% plant agar, 2.2 g l^−1^ Murashige and Skoog medium + vitamins, 0.5 g l^−1^ MES, pH 5.8; Duchefa) after HCl‐surface sterilisation and overnight stratification in 0.1% agarose at 4°C, except for EMS and IM DAP‐seq/RNA‐seq seeds, which were directly sown on soil. Seedlings intended for phyllotactic measurements were transferred to potting soil at 7 d post germination (dpg). Seedlings intended for SAM RNA‐seq were grown on a nylon mesh on top of ½MS medium +0.8% plant agar. Seedlings in plates were grown in long day conditions (16 h light, 8 h dark) at 22°C under Sylvania LUXLINE plus F18W/840 fluorescent tube lights (80 μmol·m^−2^·s^−1^, red : blue 1.4 : 1.0, red : far‐red 7.9 : 1.0). Plants in soil were grown in long day conditions at 22°C and 60% relative humidity under Lumeco LED Pro 1200 tube lights (150 μmol·m^−2^·s^−1^, red : blue 2.1 : 1.0, red : far‐red > 10.0 : 1.0). In the previously published analyses of *plt3 plt5 plt7‐t* (Prasad *et al*., 2011; Pinon *et al*., 2013), plants were grown at higher relative humidity (70%) and illuminated at lower light intensity by Sylvania GROLUX fluorescent tube lights (100 μmol·m^−2^·s^−1^, red : blue 1.4 : 1.0, red : far‐red > 10.0 : 1.0).

### Cloning

Unless specified otherwise, plasmids originate from the MoClo and Plant Parts kits (Weber *et al*., 2011; Engler *et al*., 2014). *pDR5::3xVENUS* was created by assembling a *pDR5rev2‐omegaTMV* fragment amplified from pMT280 (Thelander *et al*., 2019) in pICH41295 (Addgene #47997), *3xnlsVENUS* amplified from R2D2 (Addgene #61629; Liao *et al*., 2015) in pICH41308 (Addgene #47998), and pICH41421‐NOSt (Addgene #50339) in pICH47742 (Addgene #48001) through Golden Gate cloning (Engler *et al*., 2014). Thereafter, the *pDR5::3xVENUS* module was combined with a *p35S::DHFR* cassette and the linker pICH41744 (Addgene #48017) into pAGM4723 (Addgene #48015). *p35S::DHFR* was assembled in pICH47732 (Addgene #48000) from pICH51277‐p35S (Addgene #50268), pICH41414‐35St (Addgene #50337), and a *DHFR* amplicon amplified from pGreenII124 (Hellens *et al*., 2000) in pICH41308. The construct was inserted in the *plt3 plt5 plt7 pin1* ^*T600I*^ genome through Agrobacterium C58C1 pMP90 floral dip (Clough & Bent, 1998). The DAP‐seq constructs pSPUTK‐GG 3xFLAG‐PLT3 and pSPUTK‐GG 3xFLAG‐GFP were described previously (Kerstens *et al*., 2024). Similar constructs were assembled for *PLT5* and *PLT7* by combining pSPUTK‐GG (Kerstens *et al*., 2024), pICSL30005 (Addgene #50299), and one BsaI‐cPLT5 amplicon or four BsaI‐cPLT7 amplicons through BsaI cloning. Primers are listed in Supporting Information Table S1.

### Confocal microscopy

IMs and epidermal cell files were collected in Renaissance (RS2200) solution (Musielak *et al*., 2015) and stored at 4°C until imaging. Tissues were imaged on a Zeiss LSM710 confocal microscope with an excitation wavelength of 405 nm for RS2200 and 514 nm for VENUS signal. Before imaging with a water‐dipping lens, IMs were positioned upright in 3% agarose and submerged in Milli‐Q water.

### EMS mutagenesis and variant mapping

Dry *plt3 plt5 plt7* seeds were soaked under continuous agitation in 0.18% EMS for 16 h. The mutagenized seeds were washed eight times with 50 ml Milli‐Q water and dried on Whatman paper. M_1_ plants were grown in pools containing five genotypes each, then selfed before screening for recessive phenotypes in the M_2_. *pin1* ^*T600I*^ was identified by sequencing (150 bp paired‐end reads; NovaSeq6000; GenomeScan BV) the backcross parent and F_2_ of the first backcross (BC_1_F_2_) in two pools of 30 plants each, that is, a no‐phenotype (‘pheno‐’) pool containing wild‐type (WT) and heterozygous seedlings, and a ‘pheno+’ pool with only homozygous mutants. Reads were mapped to the TAIR10 reference genome with BWA‐MEM v.0.7.17 (Li, 2013), and variant calling was performed with GATK v.4 (McKenna *et al*., 2010) using its recommended basic filter settings. After subtraction of backcross parent variants, the following criteria for causal variant detection were used: biallelic variants only, allele depth 10–500 (median depth = *c*. 100), WT allele frequency in the ‘pheno−’ pool 0.30–0.90 (expected: 0.67) and < .05 in the ‘pheno+’ pool (expected: 0.00). BC_2_F_2_ plants were used for phenotyping.

### Plant measurements

Rosette phyllotaxis was measured from top‐view photographs of 4‐wk‐old rosettes using the angle tool in Fiji (Schindelin *et al*., 2012). For quantifying inflorescence phyllotaxis, bolts were guided upwards by sticks protruding 32 cm from the soil level and wooden clothespins, taking care to avoid twisting around the stick. Same‐age inflorescence phyllotaxis and internode length were measured in same‐age (7‐ to 8‐wk‐old plants > 32 cm) or same‐height (32 cm) plants, after removal of the top *c*. 3 cm. Internodes were measured with a ruler or digitally from scanned images. Phyllotaxis was measured with a 360° protractor. Chirality was attributed to an inflorescence based on the most frequent direction; plants without dominant chirality were discarded. Chirality was assigned manually to *tor2* ^*T56I*^ due to its extreme counterclockwise torsion. When plotting divergence angle against internode length, M‐shaped motif permutations (Besnard *et al*., 2014) were removed from the data based on the criterion: angle *i* ≥ 202.5°, angle *i* + 1 > 180°, angle *i* + 2 ≥ 202.5°. IM area was measured by making top‐down maximum intensity projections of confocal z‐stacks through the IM, then quantified manually by using the polygon selection tool in Fiji. The IM area was defined as the projected area not belonging to morphologically distinguishable flower primordia. Phyllotaxis at the IM was measured with the angle tool in Fiji, using the intersection of lines drawn straight through the middle of four to six primordia to determine the IM centre. Torsion was measured on confocal images of epidermal cell files of 7‐ to 8‐wk‐old inflorescences. Images were rotated to be parallel to the vertical axis of the stem, after which torsion was quantified with the angle tool in five nonadjacent cell files and averaged per image.

### Statistics

For pairwise comparisons, the significance of normally distributed groups was determined with Welch's *t*‐tests (assuming unequal variances), and that of non‐normally distributed groups with Wilcoxon rank‐sum tests. Percentages and proportions were compared with *z*‐tests (with ‘Yates’ continuity correction). Multiple testing corrections were performed using the Benjamini‐Hochberg method. ANOVA was used to compare multiple normally distributed groups, succeeded by Tukey's *post hoc* tests. Testing direction (i.e. left‐tailed, right‐tailed, or two‐tailed) was selected as the best suitable for each null hypothesis.

### RNA sequencing

RNA‐seq was carried out in five replicates of *c*. 50 SAMs and *c*. 50 IMs on 5 separate days within the same 40‐min window (20 replicates per min). SAMs from 7 dpg seedlings were manually dissected by cutting off the cotyledons, the two first true leaves and most of the hypocotyl; IMs were dissected from 31 to 33 dpg inflorescences, removing flowers and flower primordia until *c*. 10 primordia were left. Tissue was collected in 200 μl RNAlater (Invitrogen) on ice, which was removed after adding 750 μl Milli‐Q water before freezing in liquid N_2_. RNA was extracted with the LogSpin protocol (Yaffe *et al*., 2012), treated with DNaseI (Qiagen), then purified with EtOH purification. The libraries were sequenced on a NovaSeqX Plus at GenomeScan BV. Data analysis was performed by trimming the reads with fastp v.0.23.4 (Chen *et al*., 2018), then pseudo‐aligning to the TAIR10.1 cDNA transcriptome with kallisto v.0.46.1 (Bray *et al*., 2016). Differentially expressed genes (DEGs) were determined using DESeq2 v.1.42.1 (Love *et al*., 2014) with the standard settings, keeping genes with at least 10 counts across all five replicates, specifying harvest week covariate in the model, and defined as genes with > 25% increased or reduced expression. Gene counts were normalised visualised with the plotCounts() function.

### DAP sequencing

DAP‐seq was carried out in two replicates of *c*. 500 SAMs and *c*. 500 IMs each. SAMs from 9 dpg seedlings were dissected as described above. IMs from 4‐ to 8‐wk‐old inflorescences were dissected as described above. Tissues were collected on ice and snap‐frozen in liquid N_2_. DAP was carried out as described previously (Kerstens *et al*., 2024) and sequenced on a NovaSeq6000 platform at GenomeScan BV. Analysis was carried out as described previously, with modifications. Peaks called were called with MACS3 (Zhang *et al*., 2008) at a q‐value threshold of 0.1 for all individual samples, merging peaks within 100 bp (‐‐max‐gap 100). The peaks identified in individual replicates were then subjected to Irreproducible Discovery Rate (IDR) analysis (https://github.com/nboley/idr; v.2.0.3), retaining reproducible peaks with q‐values below 0.05. Peaks overlapping at least 1 bp with a GFP DAP peak were removed. Fragments of reads in peaks (FRiP) were calculated by dividing the number of filtered, mapped reads within peaks by the total number of filtered, mapped reads per sample. For peak overlap between data sets, peaks required at least 50% reciprocal overlap to be counted. Motif analysis was carried out on peak summits flanked by 50 bp with MEME‐ChIP (Machanick & Bailey, 2011), specifying at maximum motif width of 16.

### Phyllotactic simulations

Phyllotactic simulations relied on the trigonometric representation of the stem as proposed previously (Landrein *et al*., 2013):

(Eqn 1) $$\delta_{f}=\delta_{i}+\tan\left(\alpha \right)\;I/r$$

In which δ_f_ is the final divergence angle, δ_i_ the initial divergence angle at the IM, α the torsion angle, *I* internode length, and *r* stem radius. *In silico* Col‐0 and *plt3 plt5 plt7* inflorescences, each containing 30 internodes, were generated as follows: each internode was assigned an internode length (*I*) based on experimental data from a pseudo‐normal distribution (prohibiting negative values), using the mean and SD per position. Stem radius (*r*) was derived from a quadratic fit of experimental data, setting the maximum position to 35, then retaining the first 30 values per genotype to account for removing the top 3 cm of the bolt before measuring phyllotaxis. IM divergence angles (δ_i_) were derived from experimental data of young (*c*. 0.5–3.0 cm) IMs, assigning an initial divergence angle from a pseudo‐normal distribution to each internode. Torsion angles per internode (α) were drawn from the same normal distribution for both Col‐0 and *plt3 plt5 plt7*, pooling torsion measurements from both lines. The IM divergence angles (δ_i_) were then modified as described in Eqn 1 and binned in the same way as the experimental inflorescence data.

## Results

### PLETHORAs increase pattern robustness of inflorescence phyllotaxis

**Fig. 1:**
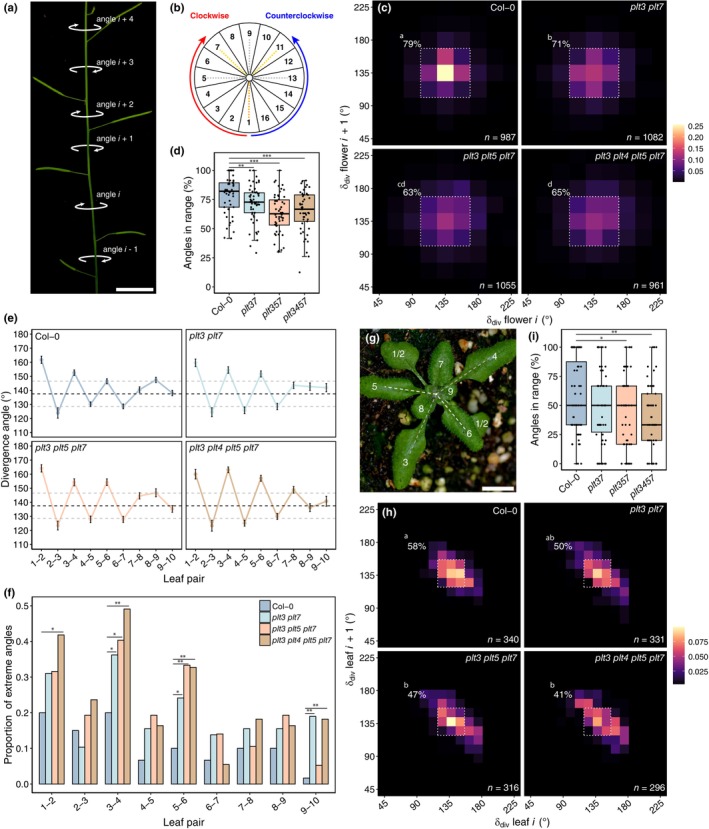
Loss of PLETHORAs (PLTs) in the Arabidopsis inflorescence and rosette reduces phyllotactic regularity. (a) Divergence angles on a clockwise‐turning Col‐0 inflorescence exemplifying angles relative to an arbitrary angle *i*. Bar, 1 cm. (b) Angle bin measurement tool, with each bin encompassing a 22.5° range. Yellow dashed lines indicate the bin containing the golden angle for clockwise (7) or counterclockwise (11) turning inflorescences. Dashed grey lines are multiples of 90°. (c) 2D‐binning heatmap showing regularity of the divergence angles (δ_div_) between two successive flowers, in which angles (*i*, x‐axis) are plotted against their subsequent angle (*i* + 1, y‐axis), with colours denoting density. The dashed square and percentage correspond to subsequent divergence angles within a 112.5–157.5° bin range. (d) Per‐plant percentage of inflorescence angles within the dashed box in (c) in Col‐0 (*n* = 50), *plt3 plt7* (*n* = 54, *P* = 0.006), *plt3 plt5 plt7* (*n* = 54, *P* = 1.3e−5), and *plt3 plt4 plt5 plt7* (*n* = 49, *P* = 5.3e−5). Significance: **, *P* < 0.01; ***, *P* < 0.001. (e) Average divergence angle (±SEM) per rosette leaf pair in Col‐0 (*n* = 60), *plt3 plt7* (*n* = 58), *plt3 plt5 plt7* (*n* = 57), and *plt3 plt4 plt5 plt7* (*n* = 55). The dashed black line denotes the golden angle, the grey dashed lines 128.5° and 146.5°. (f) Proportion of extreme δ_div_ per rosette leaf pair (δ_div_ < 110°, δ_div_ > 165°). Significance: *, *P* < 0.05; **, *P* < 0.01. (g) Counterclockwise‐turning Col‐0 rosette illustrating δ_div_ between leaf pairs 3–4 (grey lines) and 5–6 (white lines). Bar, 1 cm. (h) 2D‐binning heatmap (12.5° per bin) showing regularity of δ_div_ between successive rosette leaves. The dashed square and percentage correspond to δ_div_ within a 118.75–156.25° bin range. (i) Per‐plant percentage of rosette angles within the dashed box in (h) in Col‐0, *plt3 plt7* (*P* = 0.093), *plt3 plt5 plt7* (*P* = 0.049), and *plt3 plt4 plt5 plt7* (*P* = 0.009). Significance: *, *P* < 0.05; **, *P* < 0.01. Sample sizes as in (e). Statistical tests are all‐vs‐all (c, h) and vs Col‐0 (f) pairwise two‐proportion *z*‐tests with Benjamini‐Hochberg (BH) correction, and left‐tailed pairwise Wilcoxon rank‐sum tests with BH correction in (d) and (i). Boxplots display the minimum (lower whisker), first quartile, median (horizontal line), third quartile, and maximum (upper whisker) of the data. Outliers (> 1.5× interquartile range from hinge) are plotted individually.

We previously described that inflorescences of the *plt3 plt5 plt7* T‐DNA mutant (*plt3 plt5 plt7‐t*) in a mixed background (Col‐0 × Col‐3) adopted metastable nonspiral phyllotactic patterns (Prasad *et al*., 2011; Pinon *et al*., 2013). After switching to new growth conditions involving an upgrade from fluorescent tube to LED lights, however, the mutant only exhibited elevated deviation from the golden angle (Fig. S1), suggesting a considerable impact of environmental conditions on the phenotype and showing that our understanding of PLT‐mediated regulation of phyllotaxis was incomplete. We therefore aimed to reanalyse phyllotaxis of *plt* CRISPR mutants in a single ecotype background (Col‐0) under these conditions, thereby avoiding background effects and T‐DNA interactions. Using a 2D‐binning approach on *c*. 1000 bulked divergence angles derived from *c*. 50 7‐ to 8‐wk‐old inflorescences (Fig. 1a,b), we observed that Col‐0 plants displayed a spiral phyllotactic pattern, with the most abundant bin being 135 ± 11.25° (Fig. 1c). Deviations from the canonical pattern were present but largely limited to the surrounding 112.5 ± 11.25° and 157.5 ± 11.25° bins, and together these comprised 79% of the observed angle pairs (Fig. 1c). We then compared this phyllotactic pattern to *plt3 plt7*, *plt3 plt5 plt7*, and *plt3 plt4 plt5 plt7*, the latter of which should phenocopy the triple mutant since *PLT4* is not expressed in the IM. In each mutant, the overall phyllotactic pattern was spiral, but deviations from this pattern became apparent, which were most pronounced in the triple and quadruple mutant (Fig. 1c). Accordingly, the percentage of subsequent angle pairs within the 112.5–157.5° bin range in individual plants was reduced in each mutant (Fig. 1d). In none of the *plt* lines, however, metastable phyllotactic conformations were adopted. Thus, in our current growth conditions, loss of PLTs in the IM reduces the robustness of the phyllotactic spiral.

### PLETHORAs facilitate convergence to the golden angle in the rosette

Previously, it was demonstrated that *plt3 plt5 plt7‐t* rosette leaves could occasionally maintain a decussate arrangement (Prasad *et al*., 2011). To study this process more quantitatively, we measured divergence angles between the first 10 true leaves in Col‐0 and the three CRISPR mutants (Fig. 1e). Although all genotypes displayed damped oscillations around the golden angle, average oscillation amplitudes appeared larger in the *plt* mutants, especially at leaf pairs 5–6, with *plt3 plt5 plt7* and *plt3 plt4 plt5 plt7* having the largest effect (Fig. 1e). Moreover, we noticed that the percentage of ‘extreme’ angles, here defined as angles deviating > 27.5° from the golden angle, occurred more frequently in leaf pairs 3–4 and 5–6 of each *plt* mutant compared to the WT (Fig. 1f,g). Accordingly, overall rosette phyllotaxis (through summing angles of leaves 3–10) was also subtly noisier compared to the WT: 58% of subsequent rosette leaf pairs fell within a 118.75–156.25° range in Col‐0, but this percentage dropped to 50%, 47%, and 41% in *plt3 plt7 plt3 plt5 plt7* and *plt3 plt4 plt5 plt7*, respectively, which was statistically supported on a per‐plant level in the latter two mutants (Fig. 1h,i). We conclude that PLTs in the vegetative SAM aid in establishing regularity of leaf primordium inception position and convergence to the golden angle.

### PLETHORAs regulate primordium patterning through modulation of hormonal networks

**Fig. 2:**
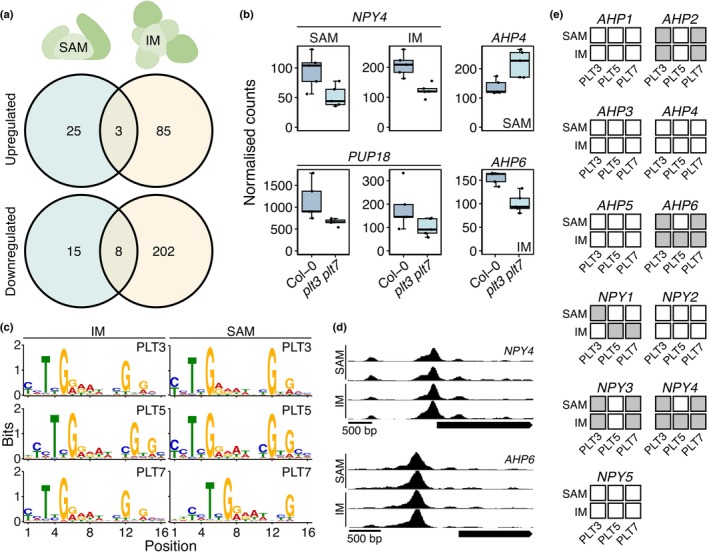
PLETHORAs (PLT) regulate *AHP* and *NPY* genes in Arabidopsis. (a) Upregulated (upper) and downregulated (lower) differentially expressed genes identified in the shoot apical meristem (SAM) (left) and IM (right). (b) DESeq2‐normalised counts of *NPY4*, *PUP18*, *AHP4*, and *HAP* in Col‐0 and *plt3 plt7*. (c) Top identified DNA affinity purification (DAP)‐seq motif for PLT3, PLT5, and PLT7 in SAMs and inflorescence meristem (IMs). (d) PLT7 DAP‐seq coverage tracks of *NPY4* and *AHP6*, with two replicates for both the SAM and IM DNA libraries. The pointed rectangle corresponds to the respective representative gene model. (e) Overview of *AHP* and *NPY* family genes bound (grey) or not bound (white) by PLTs within a [−1500 bp, 500 bp] range. Boxplots display the minimum (lower whisker), first quartile, median (horizontal line), third quartile, and maximum (upper whisker) of the data. Outliers (>1.5× interquartile range from hinge) are plotted individually.

Given the small but measurable level of noise caused by loss of PLTs, we further investigated how PLTs confer robustness to primordium inception patterns. We performed RNA‐seq of SAMs and IMs of *plt3 plt7* mutants because this mutant already displayed a minor robustness phenotype. Meristematic transcriptomes were very similar between Col‐0 and *plt3 plt7*, with only 51 and 298 DEGs identified in the SAM and IM, respectively, in line with the subtle phenotype of *plt3 plt7* (Fig. S2, Table S2). Since *PLT3* and *PLT7* are also expressed in developing leaves and flowers (Nole‐Wilson *et al*., 2005), we first examined the shared DEGs between both tissues in greater detail (Fig. 2a). Within the eight shared downregulated DEGs, we noticed the presence of a *PURINE PERMEASE* family gene (*PUP18*) and a *NAKED PINS IN YUC MUTANTS* family gene (*NPY4*), which are cytokinin transporters and regulators of PIN1 polarization, respectively (Fig. 2b, Cheng *et al*., 2007, 2008; Furutani *et al*., 2014; Hu *et al*., 2023). These data further implicate a connection of PLTs with auxin networks in the SAM and IM but also reveal a potential connection with cytokinin signalling. In congruence with this notion, we found that expression of *HISTIDINE PHOSPHOTRANFER PROTEIN 6* (*AHP6*), which channels primordium inception position through the establishment of cytokinin inhibitory fields and prevents organ co‐initiation, was reduced in *plt3 plt7* IMs (Fig. 2b, Besnard *et al*., 2014). In addition, SAM‐expressed *AHP4* expression was elevated in the *plt3 plt7* (Fig. 2b).

In order to assess whether the genes identified in our RNA‐seq could be directly regulated by PLTs, we performed DAP‐seq on SAM and IM genomic libraries using 3xFLAG‐PLT3, 3xFLAG‐PLT5, and 3xFLAG‐PLT7 baits. Importantly, using these libraries ensures retention of tissue‐specific DNA methylation marks that can influence TF binding, thus increasing peak credibility in the SAM or IM context. From integration of two biological replicates for both SAM and IM, each containing roughly 500 meristems, we identified *c*. 20 000 PLT7 peaks, *c*. 15 000 PLT3 peaks, and *c*. 600 to *c*. 2000 PLT5 peaks, revealing a much larger array of *in vitro* binding sites than observed previously (Table S3) (O'Malley *et al*., 2016; Santuari *et al*., 2016; Kerstens *et al*., 2024). In each dataset, DAP peaks occurred preferentially close to the transcription start site (Table S4, Fig. S3a), and the canonical PLT‐binding motif (Santuari *et al*., 2016; Kerstens *et al*., 2024) was strongly enriched in the peak summits (Fig. 2c). The overlap between peaks of individual PLTs exhibited a distinctly nested structure, in which PLTs for which we identified large peak numbers (e.g. PLT7) bound to nearly all peak sites identified in smaller datasets (e.g. PLT5). For instance, the great majority of PLT3 (IM: 90%; SAM: 87%) and PLT5 peaks (IM: 89%; SAM: 87%) were included in the PLT7 dataset (Table S5). Peaks were predominantly shared between SAM and IM for each PLT, again showing a nested structure when comparing larger to smaller datasets (Table S5). Across all libraries, we noticed that the average fragment‐of‐reads‐in‐peaks (FRiP) score – an empirical proxy for signal‐to‐noise ratio – correlated strongly with the number of identified peaks in each dataset (*R* ^2^ = 0.97). FRiP scores were highest for PLT7 (0.30–0.41), intermediate for PLT3 (0.18–0.24), and lowest for PLT5 (0.03–0.04), despite similar *in vitro* translation levels, perhaps reflecting differential binding affinity (Fig. S3b,c; Table S3). We thus conclude that PLT3, PLT5, and PLT7 recognize the same DNA‐binding motifs, which agrees with previous studies (Santuari *et al*., 2016; Kerstens *et al*., 2024), and these sites are generally bound by multiple PLTs in both SAMs and IMs *in vitro*.

We then focussed on peaks occurring upstream of the selected DEGs. *NPY4* was bound *c*. 50 bp upstream by all PLTs in the IM, and by PLT3 and PLT7 in the SAM (Fig. 2d,e). Likewise, we detected peaks *c*. 800 bp upstream of *AHP6* in both meristem types (Fig. 2d,e), suggesting that differential expression of *NPY4* and *AHP6* results directly from reduced PLT levels. We did not detect evidence that *PUP18* or *AHP4* were directly regulated by PLTs (Figs 2e, S3d). We next extended the analysis to other genes by screening the 1500 bp upstream and 500 bp downstream from the TSS for the presence of DAP peaks. In addition to *NPY4* and *AHP6*, we observed peaks near *NPY1*, *NPY3*, and *AHP2*, reinforcing the notion that PLTs regulate auxin and cytokinin signalling in the SAM and IM, which was further supported by the presence of peaks near the TSS of *PIN1*, *PLT5*, and *PLT7* (Figs 2e, S3e). Taken together, we hypothesize that PLTs stabilise primordium position through direct and indirect upstream regulation of auxin and cytokinin networks.

### Reduced robustness of flower primordium position cannot explain inflorescence phyllotaxis

**Fig. 3:**
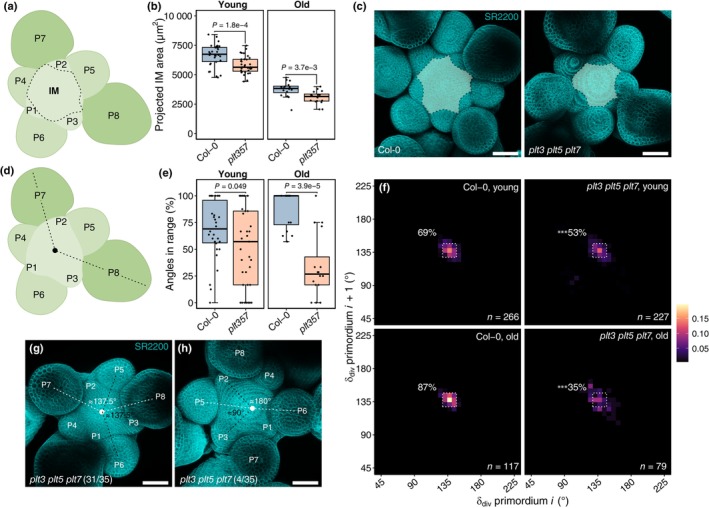
*plt3 plt5 plt7* IMs exhibit reduced organ initiation robustness in Arabidopsis. (a) Schematic overview of projected IM area measurements. (b) Projected IM area of young (*c*. 0.5–3.0 cm) and older (*c*. 32–35 cm) inflorescences in Col‐0 (*n* = 30, *n* = 16, resp.) and *plt3 plt5 plt7* (*n* = 35, *n* = 16, resp.). *P*‐values are two‐tailed Welch's *t*‐tests. (c) Example areas (highlighted) of young Col‐0 and *plt3 plt5 plt7* IMs. (d) Schematic overview of divergence angle (δ_div_) measurements between flower primordia. (e) Per‐IM subsequent angles within a 128–147° bin range. *P*‐values are left‐tailed Wilcoxon rank‐sum tests. Sample sizes are as in (b). (f) 2D‐binning heatmap (6.25° per bin) showing regularity of δ_div_ between successive flower primordia of young (*P* = 8.9e−14) and old (*P* = 1.5e−4) IMs. The dashed square and percentage correspond to the range in (e). Statistics are left‐tailed two‐proportion *z*‐tests. (g) Spiralling and decussate (h) IMs of *plt3 plt5 plt7*. Bars, 50 μm. Boxplots display the minimum (lower whisker), first quartile, median (horizontal line), third quartile, and maximum (upper whisker) of the data. Outliers (> 1.5× interquartile range from hinge) are plotted individually. IM, inflorescence meristem.

If circumferential patterning in *plt* mutants is noisier in the rosette and in the inflorescence, then it is conceivable that primordium position at the meristem is disturbed. It was previously shown that IM primordia of *plt3 plt5 plt7‐t* could be initiated at 90° or 180° angles (Prasad *et al*., 2011), but a quantitative comparison was lacking. Using confocal imaging of shoot apices, we first investigated if IM area was affected in *plt3 plt5 plt7* (Fig. 3a). The projected IM area, that is, the surface area derived from top‐down z‐stacks of confocal images, of the mutant was slightly smaller than that of the WT, both at young (*c*. 0.5–3.0 cm) and older (*c*. 32–35 cm) inflorescences, suggesting that the absence of PLTs reduces the spacing between successive flower primordia (Fig. 3b,c). We then asked if the robustness of primordium initiation position was reduced in the *plt3 plt5 plt7* (Fig. 3d). Whereas Col‐0 flower primordia were consistently initiated around the golden angle, with 69% (young) and 87% (old) of successive primordia diverging between 128° and 146°, *plt3 plt5 plt7* initiation robustness was reduced in both young and older IMs (Fig. 3e,f). Additionally, we observed infrequent decussate arrangements in *c*. 10% (4/35) of young IMs, but not old IMs (0/16), resulting in divergence angles approaching 90° and 180° (Fig. 3g,h). Despite this qualitative confirmation of our previously published data (Prasad *et al*., 2011; Pinon *et al*., 2013), we realized that flower primordium divergence in neither Col‐0 nor *plt3 plt5 plt7* matched the divergence angles observed in their respective inflorescence, and that the overall reduced robustness in *plt3 plt5 plt7* IMs was too small to explain the differences in phyllotaxis with Col‐0 (Fig. 1c). One or more additional factors must therefore be modifying the patterns established at the IM.

### A novel *pin1* allele supports loss of robustness in PLETHORA‐deficient inflorescence meristem

**Fig. 4:**
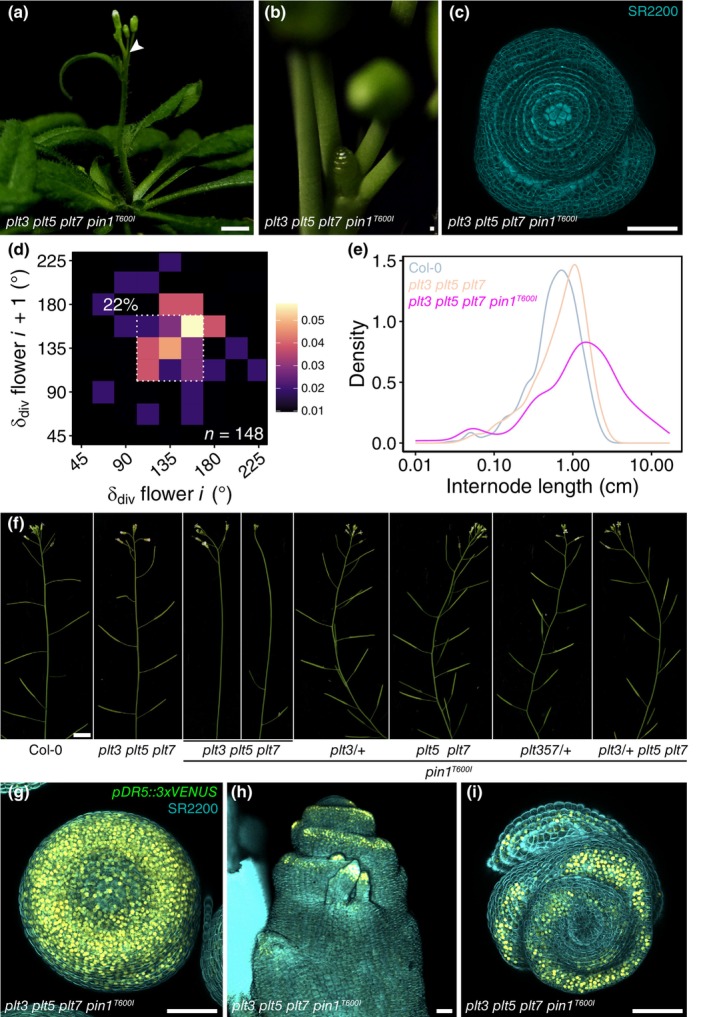
A weak *pin1* allele PLT‐dependently disrupts flower primordium inception in Arabidopsis. (a) Overview of a 5‐wk‐old *plt3 plt5 plt7 pin1* ^*T600I*^ plant. Bars, 1 cm. (b) Close‐up of an exposed IM and a maximum projection top view (c). Bars, 50 μm. (d) 2D‐binning plot of *plt3 plt5 plt7 pin1* ^*T600I*^ phyllotaxis. (e) Internode length distribution of Col‐0, *plt3 plt5 plt7*, and *plt3 plt5 plt7 pin1* ^*T600I*^. Note the logarithmic x‐axis scale. (f) F_2_ progeny of *plt3 plt5 plt7 pin1* ^*T600I*^ × Col‐0. Bars, 1 cm. (g) Expression of the auxin reporter *pDR5::3xVENUS* (nuclear) in naked or ridged *plt3 plt5 plt7 pin1* ^*T600I*^ IMs (h, i). Bars, 50 μm. IM, inflorescence meristem; PLT, PLETHORA.

If shoot and inflorescence meristems with diminished PLT3, PLT5, and PLT7 levels have reduced patterning robustness, then they should be sensitised to further perturbations. We hypothesised that additional mutations might thus allow establishment of alternative, nonspiral phyllotactic arrangements. To test this notion in an untargeted way, we performed an EMS screening in the *plt3 plt5 plt7* background. Although we were unable to identify plants from this screen with regular nonspiral phyllotaxis, we identified a mutant in which patterning robustness was strongly disturbed. This mutant failed to consistently produce lateral organs after the first *c*. 5 flowers, at which point the IMs were visible as a naked structure with or without protruding ridges (Figs 4a–c, S4). IMs did not abort completely but sporadically succeeded in the generation of fertile flowers, establishing an inflorescence with irregular phyllotaxis and extremely long internodes of up to 17 cm, with a cluster of flowers appearing at the apex during maturation (Fig. 4d,e). We then backcrossed the mutant to *plt3 plt5 plt7* and conducted whole‐genome sequencing (WGS) on the BC_1_F_2_ generation, in which the phenotype segregated (*c*. 1 : 3), indicative of a recessive allele. Mapping the causal mutation revealed only one potential candidate – a C to T substitution in exon 5 of *PIN1* – leading to a threonine to isoleucine conversion at position 600 (T600I) of the protein. The T600 residue is located in the 10th and final transmembrane segment of PIN1 and is part of the transport domain (Yang *et al*., 2022), positing that the mutant protein might be defective in auxin transport.

Interestingly, primordium formation was restored to WT levels in F_2_ progeny obtained from crossing *plt3 plt5 plt7 pin1* ^*T600I*^ BC_1_F_2_ with Col‐0 that harboured at least one functional *PLT* allele, suggesting that the observed phenotype is fully dependent on the absence of PLT3, PLT5, and PLT7 (Fig. 4f). To further investigate this dependency, we analysed the phenotype segregation ratios of progeny from *plt3 plt5 plt7 pin1* ^*T600I*^, *plt3/+ pin1* ^*T600I*^, *plt5 plt7 pin1* ^*T600I*^, and *plt3/+ plt5 plt7 pin1* ^*T600I*^ plants. In contrast to *plt3 plt5 plt7 pin1* ^*T600I*^, in which all plants (81/81) displayed the primordium initiation defects, no *plt3/+ pin1* ^*T600I*^ (0/77) or *plt5 plt7 pin1* ^*T600I*^ (0/83) were affected (Table S6). In progeny of two independent *plt3/+ plt5 plt7 pin1* ^*T600I*^ lines, the phenotype segregated roughly according to Mendelian segregation (22/125 and 30/118, resp. *c*. 18% and 25%). When genotyped (Table S7), affected plants were without exception homozygous for *plt3* (34/34), whereas plants with a WT appearance were heterozygous (49/83) or homozygous for *PLT3* (34/83).

We then introduced a *pDR5::3xVENUS* construct in the *plt3 plt5 plt7 pin1* ^*T600I*^ background to examine auxin‐induced gene activity in the ‘naked’ IMs. In IMs without protruding ridges, the 3xVENUS signal was strong throughout the entire structure, with reduced signal in the centre (Fig. 4g). In the ridged IMs, we observed high *pDR5* activity in the primordium‐like protrusions generated from the IM, but not in the centre (Fig. 4h,i). This suggests that *plt3 plt5 plt7 pin1* ^*T600I*^ IMs fail to correctly specify auxin maxima and organ initiation sites. The fact that this phenotype exclusively occurs in PLT‐deficient IMs, supports the hypothesis that PLTs confer robustness to meristems through auxin networks.

### Accelerated inflorescence development enhances *plt3 plt5 plt7* internode length

**Fig. 5:**
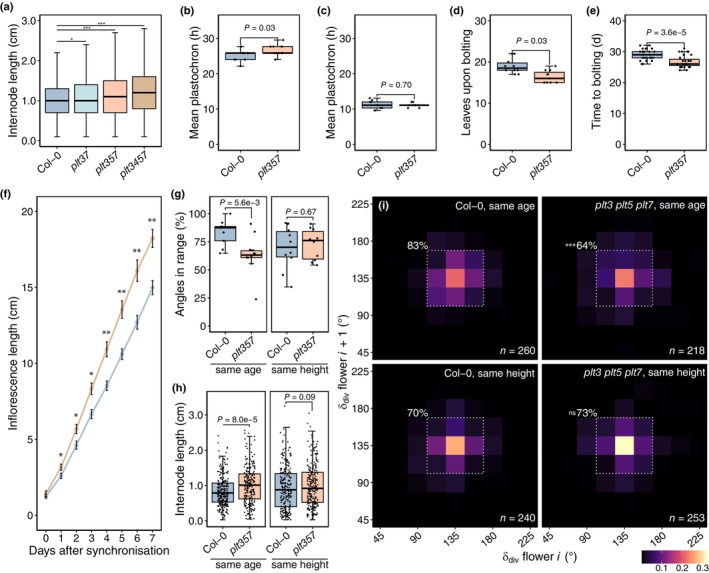
*plt* inflorescence development is accelerated in Arabidopsis. (a) Internode length of Col‐0 (*n* = 1037), *plt3 plt7* (*n* = 1136, *P* = 0.04), *plt3 plt5 plt7* (*n* = 1109, *P* = 3.0e‐4), and *plt3 plt4 plt5 plt7* (*n* = 1010, *P* = 5.4e‐10). Significance: *, *P* < 0.05; ***, *P* < 0.001. (b) Average plastochron for rosette leaves 3–15 in Col‐0 (*n* = 10) and *plt3 plt5 plt7* (*n* = 11). (c) Average plastochron in Col‐0 (*n* = 9) and *plt3 plt5 plt7* (*n* = 10) in the first 6 d after the inflorescence length exceeded 5 cm. (d) Leaves upon bolting and days to bolting (e) of Col‐0 (*n* = 10, *n* = 29, resp.) and *plt3 plt5 plt7* (*n* = 11, *n* = 31, resp.). (f) Average Col‐0 (*n* = 9) and *plt3 plt5 plt7* (*n* = 10) inflorescence length (±SEM) after synchronisation to *c*. 1.5 cm (day 0). Significance: *, *P* < 0.05; **, *P* < 0.01. (g) Per‐plant subsequent angles within a 112.5–157.5° bin range for same‐age (*n* = 9, *n* = 10, resp.) and same height (*n* = 10, *n* = 10, resp.) Col‐0 and *plt3 plt5 plt7* inflorescences (h) Internode length of same‐age (*n* = 269, *n* = 228, resp.) and same height (*n* = 250, *n* = 263, resp.) Col‐0 and *plt3 plt5 plt7* inflorescences. (i) 2D‐binning heatmap showing regularity of divergence angles (δ_div_) between successive same‐age (*P* = 2.1e−6) and same height (*P* = 0.75) flowers. The dashed square and percentage correspond to the range in Fig. 1c. Statistics are right‐tailed (a, h) and two‐tailed (c–f) Wilcoxon rank‐sum tests, left‐tailed (g) and two‐tailed Welch's *t*‐tests (b), and left‐tailed *z*‐tests (i). A Benjamini‐Hochberg (BH) correction was applied in panels (a) and (f). Boxplots display the minimum (lower whisker), first quartile, median (horizontal line), third quartile, and maximum (upper whisker) of the data. Outliers (>1.5× interquartile range from hinge) are plotted individually.

In addition to circumferential patterning, inflorescence phyllotaxis has a vertical component: the time between inception of successive primordia, or plastochron, and spacing through elongation of internodes. We therefore wondered whether these components are affected by loss of PLTs and could potentially explain the discrepancy between *plt3‐*, *plt5‐*, and *plt7*‐dependent noise observed in IMs and inflorescences. We observed that the internode length of 7‐ to 8‐wk‐old *plt3 plt7*, *plt3 plt5 plt7* and *plt3 plt4 plt5 plt7* inflorescences was subtly increased compared to WT (Fig. 5a), which we could consistently replicate for *plt3 plt5 plt7* and *plt3 plt5 plt7‐t* in independent experiments (Fig. S5a,b). To deconvolute the factors determining increased internode length in *plt* mutants, we first asked if the plastochron in *plt3 plt5 plt7* was longer than in Col‐0. Although the average plastochron in the rosette was slightly longer in the *plt* triple mutant, the plastochron in the inflorescence was identical, implying that floral organ initiation occurs at the same speed and is therefore not causal to the internode length phenotype (Fig. 5b,c). We then compared the overall bolting process between the two lines. *plt3 plt5 plt7* mutants bolted earlier, both in absolute time and with fewer rosette leaves, and the growth of the bolt was accelerated in comparison to Col‐0 after synchronisation to the same starting height (Figs 5d–f, S5c). Thus, same‐age inflorescences of *plt3 plt5 plt7* are longer than Col‐0. Since basal internodes are generally longer than apical ones (Fig. S5d,e), we asked if younger bolting age and growth speed alone were sufficient to explain enhanced internode length in *plt3 plt5 plt7*. Indeed, the internode length of Col‐0 and *plt3 plt5 plt7* inflorescences grown to exactly the same height was similar, but not when measured at the same age (Fig. 5h). Unexpectedly, we noticed that although the phyllotaxis phenotype of *plt3 plt5 plt7* inflorescences recurred in same‐age inflorescences, pattern regularity resembled that of Col‐0 in same‐height inflorescences, suggesting an interaction between internode length and phyllotaxis (Fig. 5g,i). We conclude that *plt3 plt5 plt7* internodes – and likely per extension, those of *plt3 plt7* and *plt3 plt4 plt5 plt7* – are primarily longer because of accelerated growth speed and earlier bolting, which appear to modify phyllotaxis.

### Inherent stem torsion chirality‐dependently modifies the *plt* phyllotaxis phenotype

**Fig. 6:**
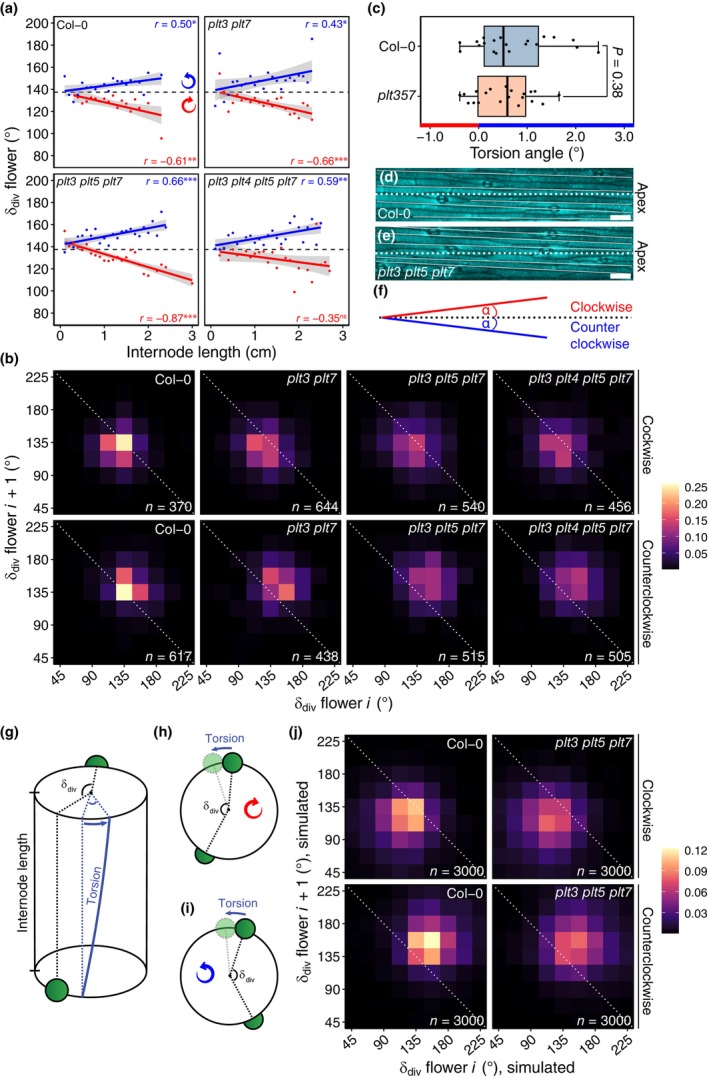
Arabidopsis Col‐0 and *plt* phyllotaxis is modified by counterclockwise torsion. (a) Divergence angle vs internode length, separated by chirality. Each dot is the average of at least four values within each 0.1 mm bin. Blue (counterclockwise) and red (clockwise) lines are linear regressions with a 95% confidence interval indicated in their respective colours. Pearson correlation coefficients for Col‐0 (*P* = 0.02, *P* = 7.6e‐3, resp.), *plt3 plt7* (*P* = 0.04, *P* = 8.2e‐4, resp.), *plt3 plt5 plt7* (*P* = 5.5e‐4, *P* = 4.3e‐8), and *plt3 plt4 plt5 plt7* (*P* = 1.8e‐3, *P* = 0.09, resp.), for counterclockwise and clockwise inflorescences, are indicated. (b) 2D‐binning heatmap showing regularity of divergence angles (δ_div_) between successive flowers, separated by chirality. A white dashed line (slope = −1) is shown for reference. (c) Measured torsion in epidermal cell files of same‐age Col‐0 (*n* = 20) and *plt3 plt5 plt7* (*n* = 20) inflorescences. *P*‐value from two‐tailed Welch's *t*‐test. Note that negative values indicate clockwise torsion, whereas positive values indicate counterclockwise torsion. Boxplot displays the minimum (left whisker), first quartile, median (vertical line), third quartile, and maximum (right whisker) of the data. All individual data points are shown. (d) Confocal top‐view (RS2200) of inflorescence epidermal cell files (and stomata) in Col‐0 and in *plt3 plt5 plt7* (e). The dashed line denotes the horizontal axis, with the inflorescence apex on the right; the solid lines correspond to cell file deflection. Bars, 50 μm. (f) Schematic view of potential clockwise and counterclockwise torsion in (d) and (e). (g) Model of how counterclockwise stem torsion affects initial phyllotactic patterning. (h) Top view of a clockwise and counterclockwise (i) meristem exhibiting counterclockwise torsion, leading to smaller and larger δ_div_ values, respectively. (j) Simulated phyllotactic patterns generated from 101 *in silico* inflorescences, with 30 internodes each, based on measured parameters for same‐age Col‐0 and *plt3 plt5 plt7* inflorescences.

To understand how increased internode length could change phyllotaxis, we tested whether divergence angle and internode length were correlated. Absolute deviations from the central 135° bin increased with longer internodes in WT plants as well as in *plt3 plt7*, *plt3 plt5 plt7*, and *plt3 plt4 plt5 plt7*, suggesting a genotype‐independent effect of internode length on divergence angle (Fig. S6). Strikingly, we noticed for each of these genotypes that the data separated into two groups when we plotted divergence angle against internode length, and that these groups corresponded to clockwise and counterclockwise turning inflorescences (Fig. 6a). Whereas divergence angles of clockwise inflorescences exhibited a negative correlation with internode length, those of counterclockwise inflorescences correlated positively (Fig. 6a). We note that the regression slopes of Col‐0 and the three *plt* mutants appeared to be similar within a 95% confidence interval (Fig. 6a). In line with this finding, phyllotactic patterns of Col‐0 and *plt* inflorescences shifted to smaller or larger divergence angles if grouped by chirality, but not in the rosette or at the IM (Fig. 6b,c). Thus, phyllotaxis in *plt* mutants is modified by internode length.

It was previously shown that a *csi1* mutant adopts a bimodal, chirality‐dependent phyllotactic pattern due to consistent counterclockwise stem twisting (Landrein *et al*., 2013), with clockwise inflorescences having divergence angles smaller than the golden angle, and counterclockwise ones having larger angles, resembling our Col‐0 and *plt* phyllotaxis patterns. To assess whether our genotypes exhibited stem twisting, we imaged the epidermal cell files of inflorescence internodes in Col‐0 and *plt3 plt5 plt7* and found that they were oriented slightly oblique (*c*. 0.6°) in both genotypes, that is, from bottom‐left to top‐right, in line with counterclockwise torsion of the stem (Fig. 6c–g). Through such torsion, longer internodes in clockwise inflorescences would cause reduced divergence angles, but increased ones in counterclockwise inflorescences (Fig. 6h,i). We thus simulated Col‐0 and *plt3 plt5 plt7* inflorescence phyllotaxis using a trigonometric representation of the stem (Landrein *et al*., 2013), through generation of *in silico* inflorescences, incorporating our measurements of primordium position (Fig. 2), per‐node internode length (Fig. S5d,e), mean torsion angle (Fig. 6c), and local stem radius (Fig. S5f,g). Our simulated inflorescences were able to replicate the phyllotaxis phenotype of same‐age *plt3 plt5 plt7* inflorescences, although chirality dependency was overestimated (Fig. 6j). Moreover, when we gradually increased or decreased *plt3 plt5 plt7* internode length and/or torsion angle in our simulations, novel phyllotactic states were obtained (Fig. S8). We conclude that, compared to same‐age WT inflorescences, phyllotaxis in *plt* mutants is modified more strongly by counterclockwise stem torsion acting on longer internodes as a consequence of accelerated bolting and growth speed.

### Stem torsion chirality‐dependently drives alternative phyllotactic patterns

**Fig. 7:**
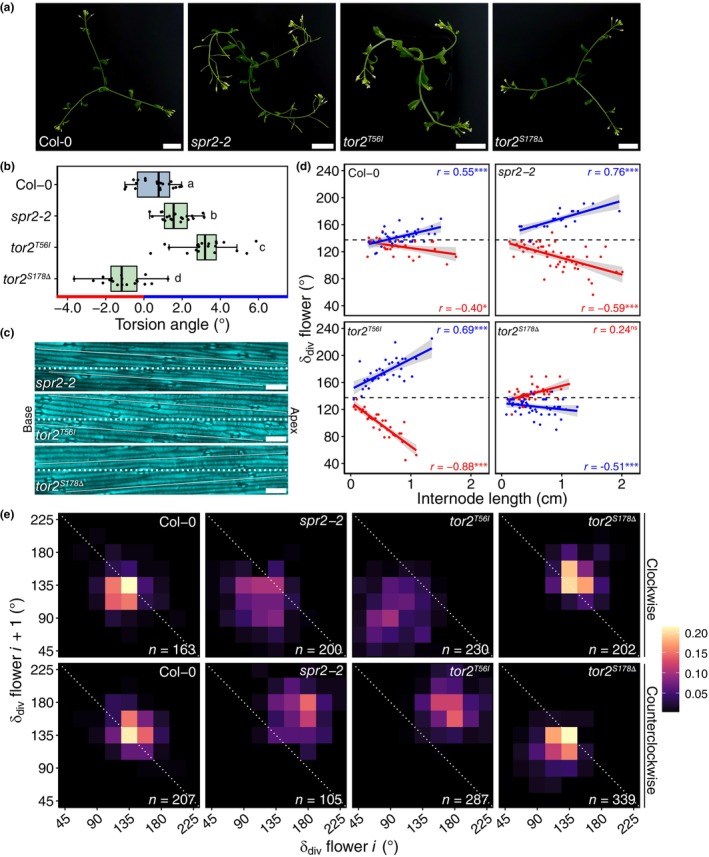
Stem torsion alters phyllotaxis chirality dependently in Arabidopsis. (a) Axillary shoots of *c*. 5‐wk‐old Col‐0 and torsion mutants, after removal of the main stem. Bars, 2 cm. (b) Measured torsion in epidermal cell files of same‐age Col‐0 and torsion mutant inflorescences (*n* = 20 each). Statistical groups (marked a, b, c and d) from an ANOVA (*P* = 1.8e‐19) followed by Tukey's *post‐hoc* tests. (c) Confocal top view (RS2200) of inflorescence epidermal cell files (and stomata). The dashed line denotes the horizontal axis, with the inflorescence apex on the right; the solid lines correspond to cell file deflection. Bars, 50 μm. (d) Divergence angle vs internode length in torsion mutants, separated by chirality, for the first 30 internodes. Each dot is the average of at least two values within each 0.01 mm bin. Blue (counterclockwise) and red (clockwise) lines are linear regressions with a 95% confidence interval indicated in their respective colors. Pearson correlation coefficients for Col‐0 (*P* = 7.2e‐4, *P* = 0.03, resp.), *spr2‐2* (*P* = 2.6e‐5, *P* = 9.8e‐6, resp.), *tor2* ^*T56I*^ (*P* = 7.1e‐6, *P* = 2.0e‐11), and *tor2* ^*S178Δ*^ (*P* = 0.10, *P* = 6.9e‐4, resp.), for counterclockwise and clockwise inflorescences, are indicated. (e) 2D‐binning heatmap showing regularity of divergence angles (δ_div_) between successive flowers, separated by chirality. A white dashed line (slope = −1) is shown for reference. Boxplots display the minimum (lower whisker), first quartile, median (horizontal line), third quartile, and maximum (upper whisker) of the data. Outliers (>1.5× interquartile range from hinge) are plotted individually.

Since the combinatorial effect of torsion and internode length on phyllotaxis was much greater than disturbed primordium position in *plt3 plt5 plt7* mutants, we decided to further verify its impact in one *spr2* and two *tortifolia2* (*tor2* ^*T56I*^ and *tor2* ^*S178Δ*^) mutants, which exhibit various degrees and directions of organ twisting (Buschmann *et al*., 2004; Ishida *et al*., 2007). Both epidermal cell files on the inflorescences and top views of the axillary shoots demonstrated that, compared to Col‐0, *tor2* ^*T56I*^ and *spr2‐2* exhibited stronger counterclockwise torsion (resp. *c*. 3.2° and *c*. 1.7°), whereas *tor2* ^*S178Δ*^ showed subtle clockwise (*c*. −1.1°) torsion (Fig. 7a–c). In line with counterclockwise stem twisting, *spr2‐2* and *tor2* ^*T56I*^ divergence angles scaled with internode length in the same way as Col‐0, but the relationship was much steeper (Fig. 7d). Inversely, the chirality dependency in *tor2* ^*S178Δ*^ was flipped due to its clockwise torsion; that is, clockwise inflorescences produced larger divergence angles on larger internodes, and counterclockwise inflorescences smaller ones (Fig. 7d). Accordingly, we observed strong effects of torsion on phyllotaxis, with torsion direction chirality‐dependently determining the direction of phyllotactic deviation, as well as nonspiral apparent pattern attractors (Fig. 7e). For instance, *tor2* ^*T56I*^ assumes a bimodal phyllotactic pattern with 90° and 180° attractors (Fig. 7e). Together, these results highlight that PLT genes, in conjunction with internode length and stem torsion, play a central role in shaping phyllotaxis and maintaining phyllotactic robustness.

## Discussion

Here, we demonstrate that PLTs influence phyllotaxis at multiple levels in the rosette and inflorescence. We show that reducing PLT levels generates a noisier phyllotactic spiral in the inflorescence and causes a slower convergence to the golden angle in the rosette, and that complete loss of PLTs exacerbates these phenotypes (Fig. 1). We observed reduced patterning robustness at the level of the IM in *plt3 plt5 plt7* – and at low frequencies, the occurrence of 90° and 180° divergence angles (Fig. 3), likely resulting from altered modulation of auxin and cytokinin signalling genes (Figs 2, 4). Nevertheless, this phenotype is minor, and it is ultimately the cooperative effect of accelerated inflorescence development, meristem chirality, and stem torsion that manifests the patterning phenotype of same‐age inflorescences of *plt3 plt5 plt7* (Figs 5, 6). In addition, we showcase the large impact of torsion in adjusting phyllotaxis postinitiation (Fig. 7).

As our work redefines the phyllotactic phenotype components of *plt3 plt5 plt7*, it raises questions about their specific functions in the SAM and IM. This is especially relevant because, unlike zygotes, lateral root primordia, callus, and the root apical meristem, SAMs and IMs do not require PLT activity to progress development or to be maintained. Even more so, our RNA‐seq did not detect differential expression of the meristematic organisation genes *WUS*, *CLV3*, *STM*, or *CUC2*, despite *plt3 plt5 plt7* having a smaller IM area (Fig. 3b,c), suggesting that reduced PLT levels do not affect the organisation of the stem cell niche. Curiously, smaller IMs have been described to promote phyllotactic robustness (Landrein *et al*., 2015), but this is not the case in *plt3 plt5 plt7*. In that aspect, *plt3 plt5 plt7* bears a striking resemblance to the *vernalisation‐independent 3* (*vip3*) mutant, which also exhibits reduced phyllotactic regularity at the IM despite forming a smaller meristem (Fal *et al*., 2017). This RNA polymerase‐associated factor 1 complex component was shown to promote the spatial regularity of auxin response peaks, linking phyllotactic variance to modulation of the auxin response. Judging from our transcriptomics and DAP‐seq data, it is an attractive hypothesis that PLTs similarly provide patterning robustness through regulation of auxin and cytokinin pathway components. This includes the *NPY* gene family, of which multiple mutants or combinations with *yuc1* and *yuc4* bear a striking resemblance to our *plt3 plt5 plt7 pin1* ^*T600I*^ mutant (Cheng *et al*., 2007, 2008; Furutani *et al*., 2014). This notion is supported by the enhanced susceptibility of *plt* double mutants to NPA or reduced *PIN1* levels (Prasad *et al*., 2011) and the complete PLT‐dependency of the *pin1* ^*T600I*^ phenotype. In addition, reduced auxin biosynthesis in the *yuc1/+ yuc4* mutant phenocopied *plt3 plt5 plt7* (Pinon *et al*., 2013). For cytokinin, PUPs have recently been described as cytokinin transporters in the SAM and IM, with *pup7*, *pup8*, *pup21* triple mutants displaying disturbed phyllotaxis and knockdown resulting in reduced cytokinin signalling (Hu *et al*., 2023). Together with the phyllotactic stabilisation conferred by the cytokinin signalling inhibitor AHP6 (Besnard *et al*., 2014), it becomes clear that precise control of hormone fluxes is critical for robust pattern formation. Especially attractive is the fact that this mode of PLT action supports both the classical auxin maximum model (Reinhardt *et al*., 2003; Smith *et al*., 2006), as well as the more recently proposed centrifugal wave model for phyllotaxis (Galvan‐Ampudia *et al*., 2020), since disturbed hormone levels and fluxes would compromise both polarisation and impact temporal dynamics. Future research will have to solidify the role of PLTs in phyllotaxis through combined loss of meristematic PLTs and PUPs/AHPs/NPYs.

When we described that loss of PLT3, PLT5, and PLT7 manifested metastable 90° and 180° pattern attractors in the IM (Prasad *et al*., 2011; Pinon *et al*., 2013), we were unable to pinpoint why these two attractors existed. It now becomes evident that metastability can be achieved from counterclockwise torsion displacing the clockwise spiral to smaller angles and the counterclockwise spiral to larger ones. Given the dependence of displacements from the golden angle on internode length, we suspect that the discrepancy between *plt3 plt5 plt7* phenotypes after switching growth conditions arose from altered genotype‐dependent inflorescence development. For instance, the original conditions could have triggered more pronounced accelerations in bolting and inflorescence growth speed in *plt3 plt5 plt7* compared to our current conditions, which together with counterclockwise stem torsion would have led to longer internodes and larger chirality‐dependent deviations from the golden angle. Our simulations incorporating inflorescence parameters from *plt3 plt5 plt7* predicted that increased internode length (by a factor of 2) can establish apparent nonrandom phyllotactic patterns *c*. 90° (clockwise) and 180° (counterclockwise), a state that can be reached with shorter internodes in combination with enhanced stem torsion (Fig. S8). Moreover, *spr2* and *tor2* mutants experimentally verified that enhanced torsion as a consequence of cortical microtubule array skewing (Buschmann *et al*., 2004; Ishida *et al*., 2007) modifies phyllotaxis (Fig. 7e). While stronger counterclockwise torsion, as measured in Col‐0/*plt3 plt5 plt7* (0.6°), *spr2‐2* (1.7°), and *tor2* ^*T56I*^ (3.2°), resulted in progressively more chirality‐dependent phyllotactic deviation from the golden angle, clockwise torsion inversed the chirality dependency. This finding demonstrates that torsion strength and direction through cortical microtubule orientation control the degree and chirality‐dependency of postinitiation phyllotactic modification. We thereby expand on previous work in which *spr2‐2* and *csi1* were shown to twist counterclockwise, leading to bimodal phyllotaxis (Landrein *et al*., 2013).

Despite exhaustive forward mutagenesis screens in the last decades, no Arabidopsis mutants have ever been described that consistently adopt alternative (nonspiral) phyllotactic patterns. Our EMS mutagenesis screening in the sensitised *plt3 plt5 plt7* background also did not yield any mutants with regular alternative primordium initiation positions, suggesting that the loss of patterning robustness in combination with additional mutations in Arabidopsis is insufficient to facilitate a transition from the spiral configuration. Simulations by Smith *et al*. (2006) already showed that phyllotactic pattern switching demands joint tuning of molecular and morphometric parameters. For instance, the ‘simplest’ transition from a distichous to a decussate pattern required elevated auxin production, reduced PZ width, and increased CZ size (Smith *et al*., 2006). In line with this finding, the only consistent transitions between phyllotactic patterns have been reported for the maize *aberrant phyllotaxy1/2* (*abph1/2*) and rice *decussate* (*dec*) mutants, of which leaves shifted from distichous to decussate phyllotaxis patterns (Jackson & Hake, 1999; Itoh *et al*., 2012; Yang *et al*., 2015). It has been postulated that SAM enlargement in these monocot mutants – perhaps jointly with defective cytokinin and/or auxin signalling in *abph1* and *dec* – allowed switching to decussate phyllotaxis, although the precise molecular mechanisms remain unclear. The required parameters for phyllotactic pattern switching might not be achievable in Arabidopsis or other eudicot SAMs/IMs due to morphological or molecular constraints.

In a broader sense, our study emphasizes that phyllotaxis in Arabidopsis is a highly conditional phenotype that relies strongly on the sum of environmental cues and at which stage it is measured. At initiation, phyllotactic robustness has been demonstrated to depend on day length (Landrein *et al*., 2015). Postinitiation, cortical microtubule orientation (leading to torsion) is of major importance, which is in turn subject to various inputs, including light, hormones, and mechanical stress (Fischer & Schopfer, 1997; Lindeboom *et al*., 2013; Wang *et al*., 2018; Adamowski *et al*., 2019). Erratic internode elongation due to interactions between *MIR164* and *CUC2* also dramatically changed phyllotaxis of the inflorescence (Peaucelle *et al*., 2007; Sieber *et al*., 2007). Furthermore, it remains unclear to what extent SAM/IM size is influenced by bolting parameters, both in WT and mutant backgrounds – including in *plt3 plt5 plt7* – and how this interaction influences phyllotaxis. Together with our finding that reduced *plt3 plt5 plt7* IM organ initiation robustness is overshadowed by postinitiation pattern modifications, this also implies that even if novel stable phyllotactic patterns in the IM are found, it will be very challenging to maintain such patterns across an elongating plant responding to its environment. Instead, controlling internode length and torsion might offer a better alternative strategy to modify phyllotaxis, albeit dependent on meristem chirality.

## Competing interests

None declared.

## Author contributions

Conceptualization: MK, HH, BS, VW; Methodology: MK, HH, BS, VW; Formal analysis: MK, FK; Investigation: MK, FK; Writing: MK, VW; Editing: BS; Visualization: MK; Supervision: BS, VW; Project administration: VW; Funding acquisition: MK.

## Disclaimer

The New Phytologist Foundation remains neutral with regard to jurisdictional claims in maps and in any institutional affiliations.

## Supporting information


**Fig. S1** Inflorescence phyllotaxis of *plt3 plt5 plt7‐t* is destabilized compared to same‐age Col‐0 plants.
**Fig. S2** SAM and IM transcriptomes between Col‐0 and *plt3 plt7* differ slightly.
**Fig. S3** PLTs bind target genes close to the TSS.
**Fig. S4** Morphology of *plt3 plt5 plt7 pin1* ^*T600I*^ shoot apices.
**Fig. S5** *plt3 plt5 plt7* inflorescence development is accelerated.
**Fig. S6** Absolute deviation from the golden angle bin correlates positively with internode length.
**Fig. S7** Rosette and IM phyllotactic patterns are not dependent on chirality.
**Fig. S8** Increasing internode length and/or torsion angle changes phyllotactic patterning of simulated inflorescences.

**Table S1** Oligonucleotides used in this study.
**Table S2** Identified DEGs in SAMs and IMs of Col‐0 and *plt3 plt7*.
**Table S3** DAP‐seq experimental metrics.
**Table S4** DAP‐seq peak annotations relative to the TSS.
**Table S5** Overlap between DAP‐seq datasets.
**Table S6** *pin1* ^*T600I*^ phenotype segregation in *plt* mutant backgrounds.
**Table S7** *pin1* ^*T600I*^ phenotype–genotype correlation in *plt3/+ plt5 plt7*.
Please note: Wiley is not responsible for the content or functionality of any Supporting Information supplied by the authors. Any queries (other than missing material) should be directed to the *New Phytologist* Central Office.

## Data Availability

The DAP‐seq, RNA‐seq, and *pin1* ^*T600I*^ allele WGS data are available in the Gene Expression Omnibus accession numbers GSE289412, GSE289413, and GSE289414, respectively.
