# Supplementary material for: PLETHORAs shape Arabidopsis phyllotaxis through modulation of patterning robustness and accelerated inflorescence development
Source: New Phytol. 2025 Oct 11;249(1):495–511. doi: 10.1111/nph.70620 (PMC12676083; doi:10.1111/nph.70620)
Supplement: Supplementary file 1 — Fig. S1 Inflorescence phyllotaxis of plt3 plt5 plt7‐t is destabilized compared to same‐age Col‐0 plants. Fig. S2 SAM and IM transcriptomes between Col‐0 and plt3 plt7 differ slightly. Fig. S3 PLTs bind target genes close to the TSS. Fig. S4 Morphology of plt3 plt5 plt7 pin1 T600I shoot apices. Fig. S5 plt3 plt5 plt7 inflorescence development is accelerated. Fig. S6 Absolute deviation from the golden angle bin correlates positively with internode length. Fig. S7 Rosette and IM phyllotactic patterns are not dependent on chirality. Fig. S8 Increasing internode length and/or torsion angle changes phyllotactic patterning of simulated inflorescences. [file NPH-249-495-s002.pdf]

New Phytologist Supporting Information

Article title:
PLETHORAs shape Arabidopsis phyllotaxis through modulation of patterning robustness and accelerated inflorescence development

Authors:
Merijn Kerstens, Freek van der Klugt, Hugo Hofhuis, Ben Scheres and Viola Willemsen

Article acceptance date:
15 September 2025

Supporting table legends [provided as a separate Excel file]

Table S1. Oligonucleotides used in this study.

Table S2. Identified differentially expressed genes in SAMs and IMs of Arabidopsis Col-0 and *plt3 plt7*. SAM = shoot apical meristem, IM = inflorescence meristem, N/A = not available.

Table S3. DAP-seq experimental metrics**.** SAM = shoot apical meristem, IM = inflorescence meristem, IDR = irreproducible discovery rate, FRiP = fragment of reads in peaks.

Table S4. DAP-seq peak annotations relative to the closest transcription start site (TSS). SAM = shoot apical meristem, IM = inflorescence meristem.

Table S5. Overlap between DAP-seq datasets. SAM = shoot apical meristem, IM = inflorescence meristem.

Table S6. $pin1^{T600I}$ phenotype segregation in Arabidopsis *plt* mutant backgrounds.

Table S7. $pin1^{T600I}$ phenotype-genotype correlation in Arabidopsis *plt3/+ plt5 plt7*.

Supporting figures

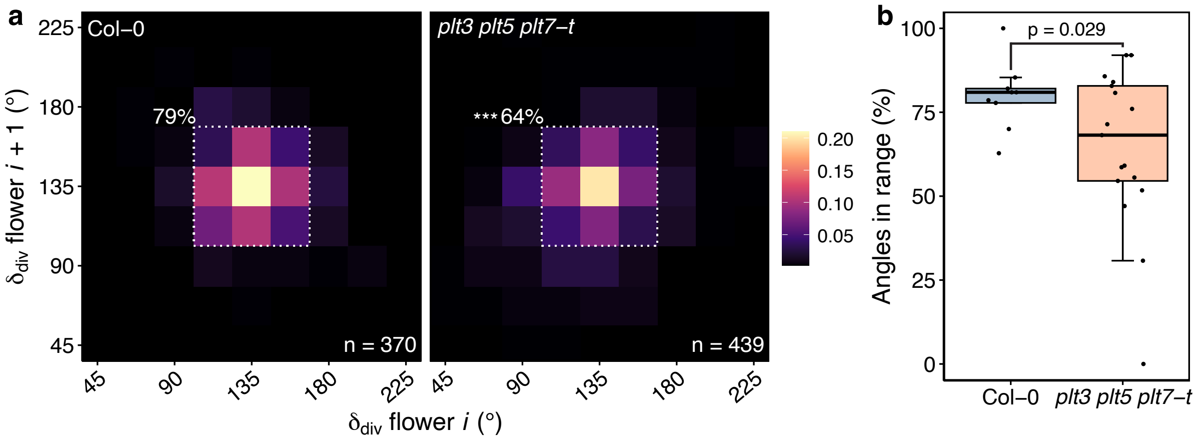

Figure S1. Inflorescence phyllotaxis of *plt3 plt5 plt7-t* is destabilized compared to same-age Col-0 plants. (**a**) 2D-binning heatmap showing regularity of the divergence angles (δ_{div}) between successive flowers, in which angles (*i*, x-axis) are plotted against their subsequent angle (*i* + 1, y-axis), with colours denoting density. The dashed square and percentage correspond to divergence angles falling within the 112.5-157.5° bin range. Statistical test is a two-tailed two-proportion z-test (***: p = 3.8e-6). (**b**) Per-plant percentage of angles within the dashed box in (**a**) in Col-0 (n = 9) and *plt3 plt5 plt7-t* (n = 17). p-value is from a two-tailed Welch's t-test. Boxplots display the minimum (lower whisker), first quartile, median (horizontal line), third quartile, and maximum (upper whisker) of the data. Outliers (>1.5x interquartile range from hinge) are plotted individually.

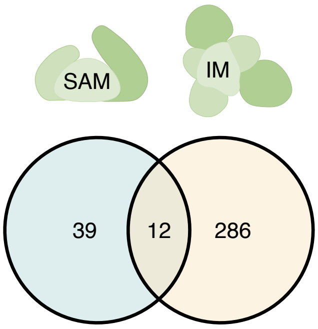

Figure S2. SAM and IM transcriptomes between Col-0 and *plt3 plt7* differ slightly. Differentially expressed genes identified in the SAM (left) and IM (right). SAM = shoot apical meristem, IM = inflorescence meristem.

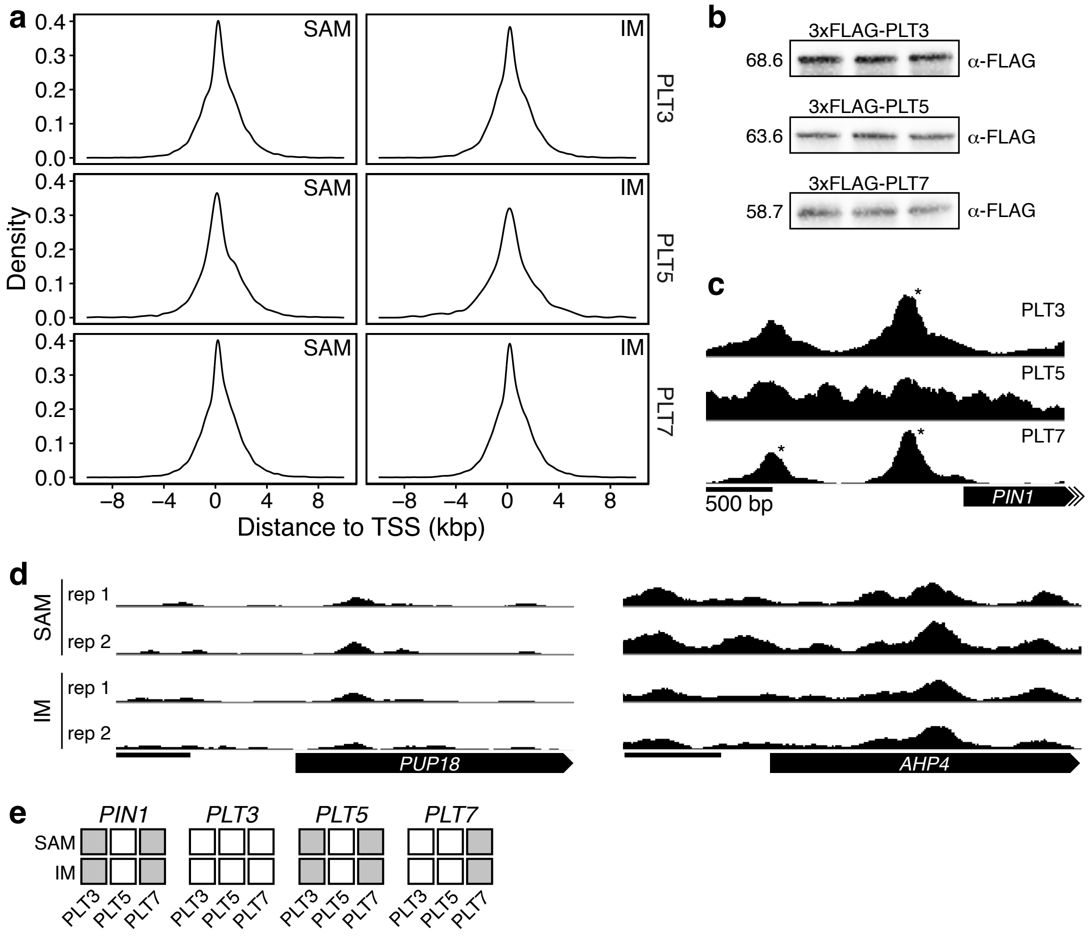

Figure S3. PLTs bind target genes close to the TSS. (**a**) Density plots of significant peak position in relation to the TSS. (**b**) Western blot of equal 3xFLAG-PLT in vitro translation reaction fractions, with three replicates each. Estimated molecular weight in kDa is indicated. (**c**) DAP-seq coverage tracks in one IM replicate showing a region upstream of *PIN1*. Asterisks indicate significant peaks after replicate integrating through IDR. * indicates a significant peak. (**d**) PLT7 DAP-seq coverage tracks of *PUP18* and *AHP4*, in which no significant peaks were found. Scale bars are 500 bp. (**e**) Overview of genes with a peak (grey) within a [-1500 bp, 500 bp] range. SAM = shoot apical meristem, IM = inflorescence meristem, DAP = DNA Affinity Purification.

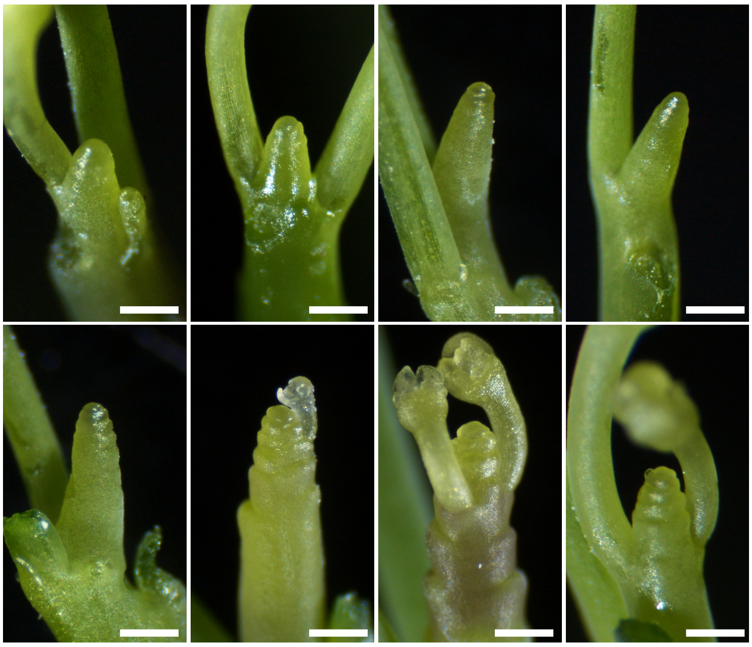

Figure S4. Morphology of *plt3 plt5 plt7 pin1*^{T600I} shoot apices. Apices are either smooth (upper) or contain ridges (lower). Scale bars are 500 µm.

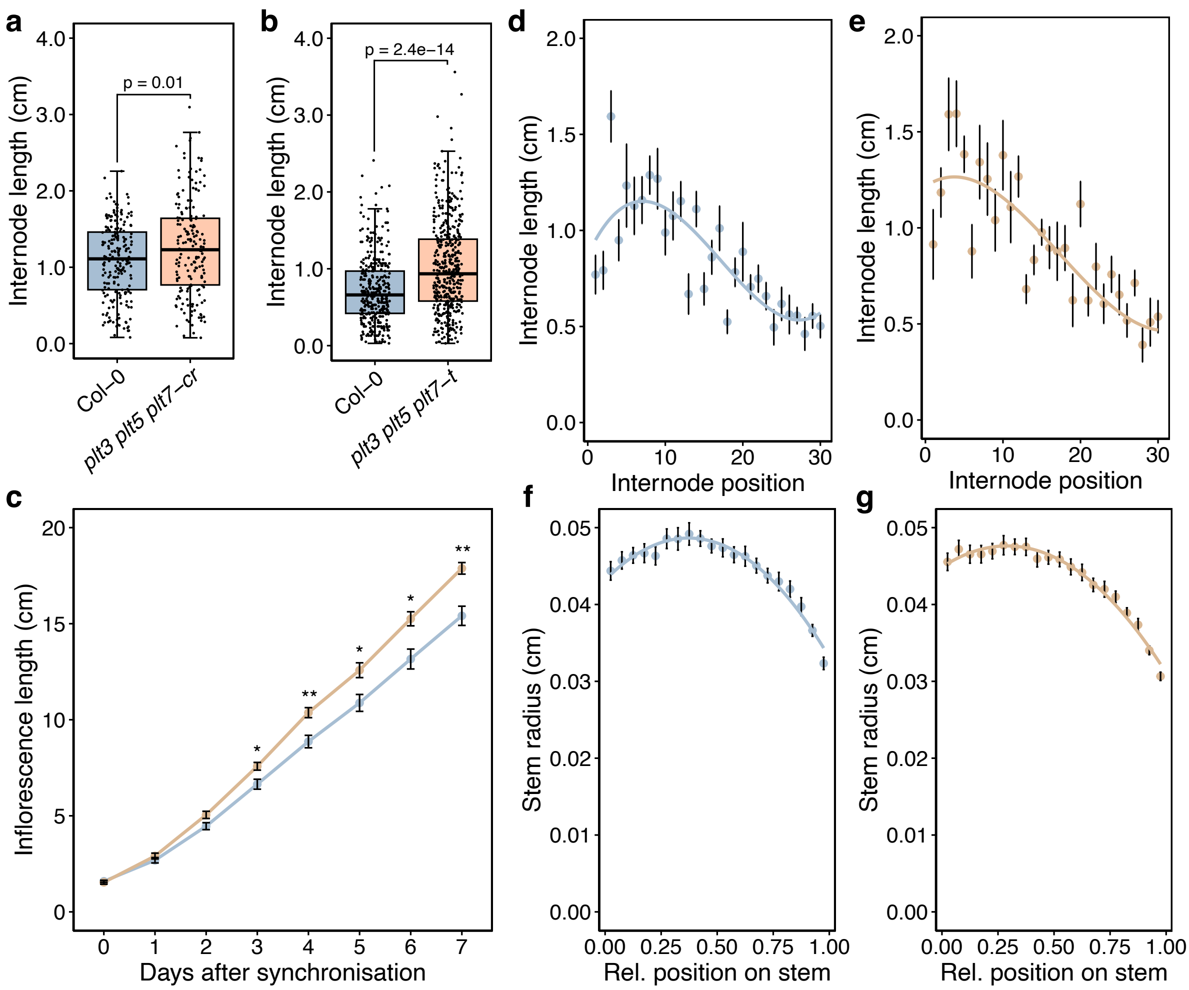

Figure S5. *plt3 plt5 plt7* inflorescence development is accelerated. (**a**) Internode length of same-age Col-0 (n = 209) and *plt3 plt5 plt7* (n = 183) inflorescences. p-value from right-tailed Wilcoxon rank-sum test. (**b**) Internode length of same-age Col-0 (n = 379) and *plt3 plt5 plt7-t* (n = 456) inflorescences. p-value from right-tailed Wilcoxon rank-sum test. (**c**) Average Col-0 (n = 8) and *plt3 plt5 plt7* (n = 8) inflorescence length (± SEM) after synchronisation to ~1.5 cm (day 0). Significance from two-tailed Wilcoxon rank-sum tests with BH correction; * $p < 0.05$, ** $p < 0.01$. (**d**) Internode length (± SEM) over internode position (base to apex), for the first 30 internodes, with third-degree polynomial fit in Col-0 (n = 9) and *plt3 plt5 plt7* (n = 10) (**e**). (**f**) Stem radius (± SEM) over relative stem position with quadratic fit in Col-0 (n = 17) and *plt3 plt5 plt7* (n = 18) (**g**). Boxplots display the minimum (lower whisker), first quartile, median (horizontal line), third quartile, and maximum (upper whisker) of the data. Outliers (>1.5x interquartile range from hinge) are plotted individually.

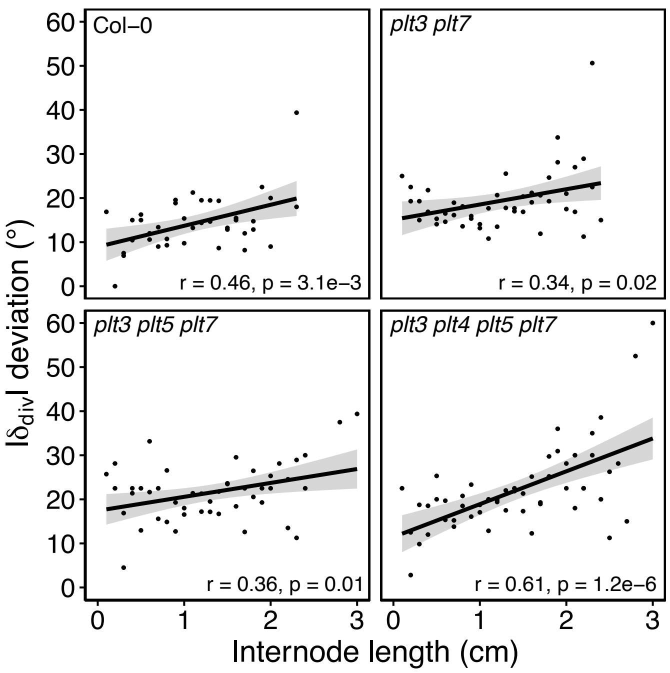

Figure S6. Absolute deviation from the golden angle bin correlates positively with internode length. Each dot is the average of at least three values within each 0.1 mm bin for internode positions 2 to 25. Black lines are linear regressions with the 95% confidence interval in grey. δ_{div} = divergence angle.

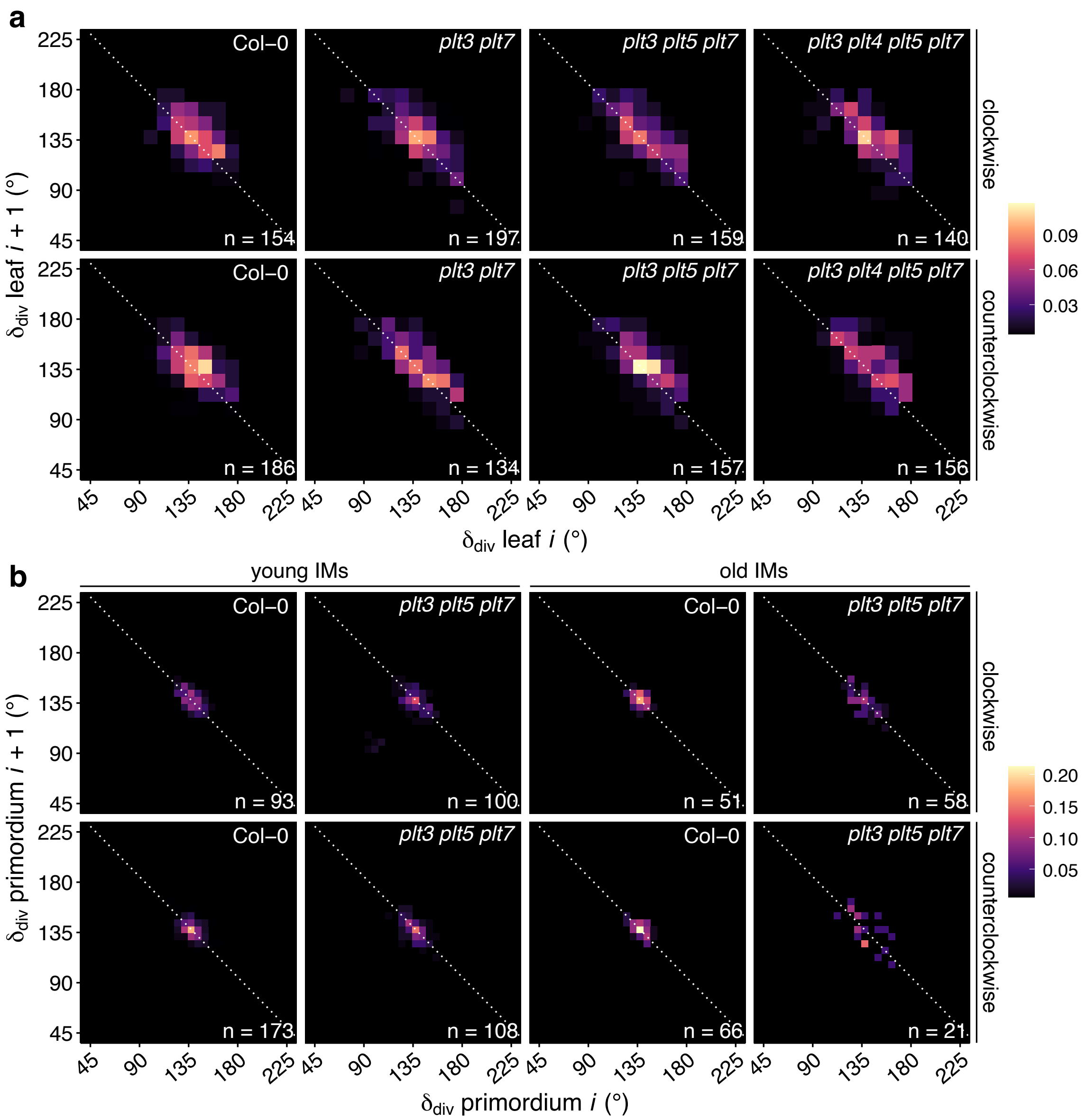

Figure S7. Rosette and IM phyllotactic patterns are not dependent on chirality. (**a**) 2D-binning heatmap (6.25° per bin) showing regularity of divergence angles (δ_{div}) between successive rosette leaves and flower primordia at the IM (**b**), separated by chirality. The white dotted line serves as a visual guide (slope = -1).

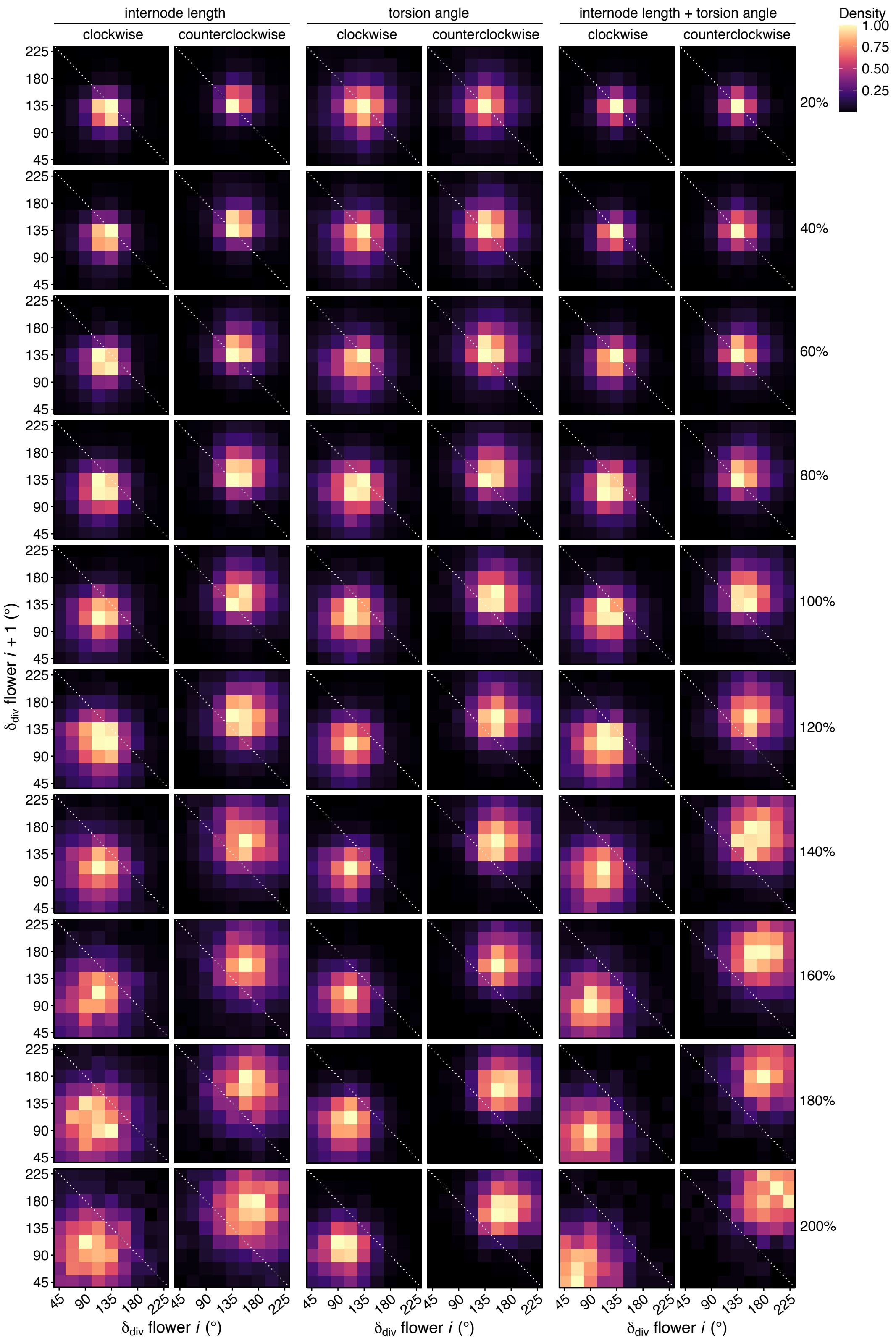

Figure S8. Increasing internode length and/or torsion angle changes phyllotactic patterning of simulated inflorescences. Clockwise and counterclockwise patterns generated from 101 in silico inflorescences, with 30 internodes each, based on measured parameters for *plt3 plt5 plt7* inflorescences as in Fig. 6j (= 100%). For phyllotactic predictions with altered internode length and/or torsion angle, only the mean values were changed in 20% increments (> 100%) or decrements (< 100%). δ_{div} = divergence angle.
